# Piloting of a Screen‑Triage‑Treat Surgical Approach Model for Management of Anal Cancer in Liberia

**DOI:** 10.5334/aogh.4576

**Published:** 2024-12-04

**Authors:** Christopher W. Reynolds, Whitney Lieb, Andrea Schecter, Michael M Gaisa, Stephen K. McGill, Evans L. Adofo, Ann Marie Beddoe

**Affiliations:** 1 Department of Surgery, University of Michigan, Ann Arbor, MI, USA; 2 Department of Obstetrics, Gynecology, and Reproductive Science, Icahn School of Medicine at Mount Sinai, New York, NY, USA; 3 Division of Infectious Diseases, Icahn School of Medicine at Mount Sinai, New York, NY, USA; 4 Marshall Minority Health Institute, Marshall University, Huntington, West Virginia, USA; 5 Stop AIDS in Liberia (SAIL), Monrovia, Montserrado, Liberia; 6 Director of Global Women’s Health, Mount Sinai Health System, New York, NY, USA

**Keywords:** Anal cancer, men who have sex with men (MSM), Liberia, surgical care, human papillomavirus

## Abstract

*Background:* While cancer is a leading cause of death worldwide, significant disparities exist in care access in low‑ and middle‑income countries (LMICs). In Liberia, screening and treatment for anal cancers remain limited, and are exacerbated among vulnerable groups, including men who have sex with men (MSM). Screen‑triage‑treat models for cancerous lesions have been successful in reducing cervical cancer mortality, but the feasibility of this approach has not been studied for anal cancers in a low‑resource context.

*Objective:* The aim of this study is to determine the feasibility of implementing a screen‑triage‑treat model for anal high‑grade squamous intraepithelial lesions (aHSIL) among MSM in Liberia.

*Methods:* This descriptive study represented a collaboration between Stop AIDS in Liberia (SAIL) and health institutions in Liberia and the USA. MSM and transgender participants were recruited through convenience sampling with SAIL peer‑educators. A survey validated by SAIL experts assessed demographics and sexual risk factors. Participants underwent anal self‑swabbing for high‑risk human papillomavirus (HPV) and offered human immunodeficiency virus (HIV) testing. Those with positive results were offered a screen‑triage‑treat model through high‑resolution anoscopy (HRA) and infrared coagulation (IRC). Data were cleaned and analyzed in SPSS.

*Findings:* Among 110 participants, most were single (*n* = 94, 88%) and without formal employment (*n* = 21, 75%). Participants engaged in regular anal (*n* = 64, 60%), oral (*n* = 62, 58%), and receptive sex (*n* = 58, 54%), and sex with women (*n* = 51, 48%). Nearly 20% of participants reported being HIV positive (*n* = 21). In all, 50 participants (45%) tested positive for anal high‑risk HPV, 34 (68%) elected to undergo HRA, and 10 (84%) were treated with IRC. Of those who underwent HRA, 75% tested HIV positive.

*Conclusions:* Our findings suggest that a screen‑triage‑treat model presents a feasible option to identify and reduce the incidence of anal cancer among MSM in Liberia. The screen‑triage‑treat model, with proven success in management of cervical dysplasia, may be a viable option to treat aHSIL for anal cancer prevention in LMICs.

## Introduction

Although commitments to health equity are gaining increasing international attention, significant disparities in access to cancer care persist around the world [1]. Despite cancer being the second leading cause of death worldwide, a significant untreated disease burden remains in low‑ and middle‑income countries (LMICs) [2]. Out of approximately 9.9 million cancer deaths in 2020, nearly 70% were in LMICs, with the most significant disparities between high‑ and low‑income countries being from preventable and treatable cancers [3, 4]. Persons with immunocompromising conditions are at increased risk of developing and experiencing significant morbidity and mortality from cancer, including those with human immunodeficiency virus (HIV). Among these cancers are those associated with high‑risk human papillomavirus (hrHPV), including cervical and anal cancer [5]. The lack of preventive care, systematic screening, and HPV vaccination programs in LMICs has led to a remarkable shift in cervical cancer incidence, leading to a disease burden of approximately 90% of worldwide cases [6].

Although deaths from acquired immunodeficiency syndrome (AIDS) are decreasing with increasing access to antiretroviral (ARV) therapy, anal cancer rates among people living with HIV/AIDS (PLWHA) are on the rise [7]. PLWHA are at disproportionately high risk for HPV‑associated anal cancer [5]. Anal high‑grade squamous intraepithelial lesions (aHSIL) are considered the immediate cancer precursors and have been reported to progress to invasive anal cancer at a rate of up to 1.9% per year [7]. Since hrHPV is causally linked to > 90% of anal cancers, utilizing this disease as a screening tool either alone or in conjunction with anal cytology has shown promise in reducing cancer morbidity [8, 9].

While anal cancer prevention programs in the USA are aimed at early detection and treatment of aHSIL and the ANCHOR trial has recently shown that this approach significantly reduces anal cancer rates, these interventions are not available in Western African countries such as Liberia [10]. Following decades of civil conflict and the 2014–2016 Ebola crisis, Liberia’s already weakened healthcare system has faced challenges in piloting and scaling cancer prevention and treatment programs. Currently, there are limited treatment options for anal cancer in Liberia, despite high rates of HIV and HPV among populations at particular risk, such as men who have sex with men (MSM), transgender persons, and sex workers [11]. While Liberia has access to HPV vaccines, uptake has been low, and there have been challenges related to sexual and health educational programming and screening and treatment of precancerous and cancerous lesions. The lack of uptake of diagnostic and therapeutic options for persons with anal cancer in Liberia results in an unknown burden of morbidity and mortality from this preventable and treatable disease.

Previous studies have shown that a screen‑triage‑treat model for cervical high‑grade squamous intraepithelial lesions (HSIL) was successful in substantially reducing morbidity and mortality of cervical cancer in low‑resource settings where histopathology is often unavailable [12]. Screening with triage prior to treatment allows for diagnosis and simultaneous treatment of lesions suspicious for HSIL via an excisional surgical or ablative procedure, bypassing the need for biopsy, pathological confirmation, and follow‑up visits. Though these programs have been effective in reducing cervical cancer and HSIL rates, no data are available on the efficacy of similar models for identifying and treating anal pre‑cancers in a low‑resource setting [13, 14]. In this descriptive study, we sought to determine whether the implementation of a similar screen‑triage‑treat model for anal HSIL was feasible among a high‑risk population of MSM in Liberia. Specifically, participants were triaged for HPV as part of a larger HPV screening program, with subsequent anoscopy for HPV positive participants.

## Methods

### Study design and participants

This non‑randomized, descriptive project was designed and completed in collaboration with Stop AIDS in Liberia (SAIL), a grassroots HIV/AIDS and LGBTQ support organization in Liberia; Redemption Hospital and John F. Kennedy Memorial Hospital in Monrovia; and the Mount Sinai Division of Global Women’s Health. It was completed as part of a larger study on HPV knowledge and prevalence [11]. This partnership developed a novel survey tool to assess participant demographics and sexual risk factors, which was content validated by staff at SAIL who identified as MSM. The survey was designed in RedCap but printed on paper to be completed with pen given the lack of accessible technology at the study site and preferences expressed by SAIL staff.

SAIL has an established network of MSM, transgender persons, sex workers, and persons living with HIV and uses a peer‑educator model to provide social and medical resources for their care. For this study, MSM and transgender participants were recruited through a convenience sampling method by SAIL peer‑educators. This methodology was considered most appropriate, as it allowed the required privacy to protect community participants and did so through a trusted source. Peer educators fully explained each component of the research study to participants who signed a written consent before participation. Participants were free to participate in only one part of the study and to renounce their participation at any time without consequences. Participants received 100 Liberian dollars, equivalent to 1 USD at the time of study, to cover transportation costs.

### Data and specimen collection

Participants first completed surveys on demographics and risk factors covering sexual histories and practices. Study investigators and peer educators were present to answer questions as participants completed the survey. Following the survey, participants received instruction from peer educators on anal self‑swabbing to assess the presence of high‑risk HPV strains. HPV testing kits were used that are validated to detect a battery of high‑risk carcinogenic HPV types, including 16, 18, 31, 33, 35, 39, 45, 51, 52, 56, 58, 59, 66, and 68, using full genome probes complementary to HPV DNA, specific antibodies, signal amplification, and chemiluminescent detection. The *careHPV* sample kit contains collection medium and self‑sampling care brushes and is ideal for low‑resource settings, given that refrigeration of accessories is not required before and up to 14 days following sample collection, and the anal swab can be completed without a trained medical provider. Self‑swabbing of the anal canal was completed in a private area by study participants, to maintain privacy, by inserting the brush 3–4 cm into the anus and rotating twice. Collection samples were immediately delivered to John F. Kennedy (JFK) Hospital, where they were stored at a refrigerated temperature and tested within 48 hours. Participants with results positive for hrHPV were contacted by peer educators and informed of their results. At this time, HIV testing was offered to all participants using rapid oral HIV antibody tests (OraQuick). Medical professionals were available to answer questions from participants.

### Screen‑Triage‑Treat model

Participants with results positive for hrHPV were offered to undergo a screen‑triage‑treat model by performing high‑resolution anoscopy (HRA) to identify lesions suspicious for aHSIL by visual appearance. The procedure was performed in a private procedure room at Redemption Hospital, following participants’ signing of a medical treatment consent form. Lesions that met criteria to warrant suspicion of aHSIL were treated with infrared coagulation (IRC). IRC uses infrared light as a heat source to coagulate vessels and is typically used in the treatment of hemorrhoids and high‑grade intraepithelial squamous lesions. The HRA and IRC treatment was performed by an infectious disease physician who is an expert in HRA and the treatment of high‑grade dysplastic lesions involving anal cancer and perianal skin. Participants were monitored for adverse reactions and followed up to assess treatment outcome. Patients whose disease was considered too extensive for IRC were treated by a visiting colorectal surgery team.

### Data analysis

Survey results were manually entered and cleaned in Microsoft Excel. Data were imported and analyzed in SPSS to calculate demographic and risk factor frequencies. Flowcharts were constructed to demonstrate the screen‑triage‑treat care pathway for study participants.

### Ethics statement

This study was performed in accordance with the principles outlined in the Declaration of Helsinki. It was also reviewed and approved by the Institutional Review Board at John F. Kennedy Medical Center, Monrovia, Liberia. Informed consent was obtained from all study subjects prior to participation.

## Results

### Demographics

A total of 110 participants were enrolled in the study, of which 107 completed a sufficient portion of the survey (> 75%; Table 1). All participants identified as men who have sex with men (MSM). Nearly 90% of participants were single (*n* = 94, 87.9%), and three‑fourths (*n* = 80, 74.8%) reported no formal employment. One in five participants self‑reported being HIV positive (*n* = 21, 19.6%) at the time of survey. Most were born in and resided in urban Monrovia, but participants hailed from multiple counties throughout Liberia. Alcohol use (*n* = 65, 60.7%) and tobacco use (*n* = 31, 29.0%) were reported, while intravenous (IV) drug use was less common but still present (*n* = 9, 8.4%). Sexual practices varied among participants. MSM respondents participated in regular anal (*n* = 64, 59.8%), oral (*n* = 62, 57.9%), and receptive sex (*n* = 58, 54.2%). High‑risk practices included having unprotected sex in the last year (*n* = 58, 54.2%), more than 20 lifetime sexual partners (*n* = 40, 37.4%), and an age of first sexual intercourse under 15 years old (*n* = 22, 20.6%). Approximately half of MSM participants (*n* = 51, 47.7%) reported having sex with women regularly.

**Table 1:** Demographic Factors and Sexual Histories.

	NUMBER	PERCENT
Total Participants	107	100%
Marital Status		
Single	94	87.9%
Married	6	5.6%
Cohabitating	4	3.7%
Divorced	3	2.8%
Religion		
Christian	102	95.3%
Muslim	3	2.8%
None	2	1.9%
Place of Birth		
Liberia	91	85.0%
Ivory Coast	3	2.8%
Ghana	2	1.9%
County of Residence*		
Urban	81	75.7%
Rural	26	24.3%
Education (highest level reached)		
None	8	7.5%
Primary	1	0.9%
Secondary	8	7.5%
High school	51	47.7%
Post‑high school	14	13.1%
College	24	22.4%
HIV tested		
Yes	83	77.6%
No	24	22.4%
Self‑reported HIV status?		
Positive	21	19.6%
Negative/unsure	86	81.4%
Alcohol use frequency (per week)		
More than 10 drinks	7	6.5%
5–10 drinks	14	13.1%
3–5 drinks	17	15.9%
1–2 drinks	28	26.2%
Shares needles when using IV drugs		
Yes	2	1.9%
No	105	98.1%
Type of anal sex		
Penetrative	41	38.3%
Receptive	26	24.3%
Both penetrative and receptive	32	29.9%
Age of first sexual intercourse		
Younger than 15 years old	22	20.6%
15–20 years old	73	68.2%
21–25 years old	11	10.3%
Older than 26 years old	1	0.9%
Unprotected sex in the last year		
More than 10 times	6	5.6%
5–10 times	7	6.5%
1–4 times	45	42.1%
Never	49	45.8%
Number of sexual partners in lifetime		
More than 20 people	40	37.4%
11–20 people	13	12.1%
5–10 people	23	21.5%
1–4 people	31	29.0%
Sexual partners in the last 6 months		
More than 20 people	4	3.7%
11–20 people	5	5.6%
5–10 people	11	10.3%
1–4 people	74	69.2%
0 people	9	8.4%
Current partner circumcised		
Yes	102	95.3%
No	4	3.7%

### Screen‑Triage‑Treat model

**Figure 1:**
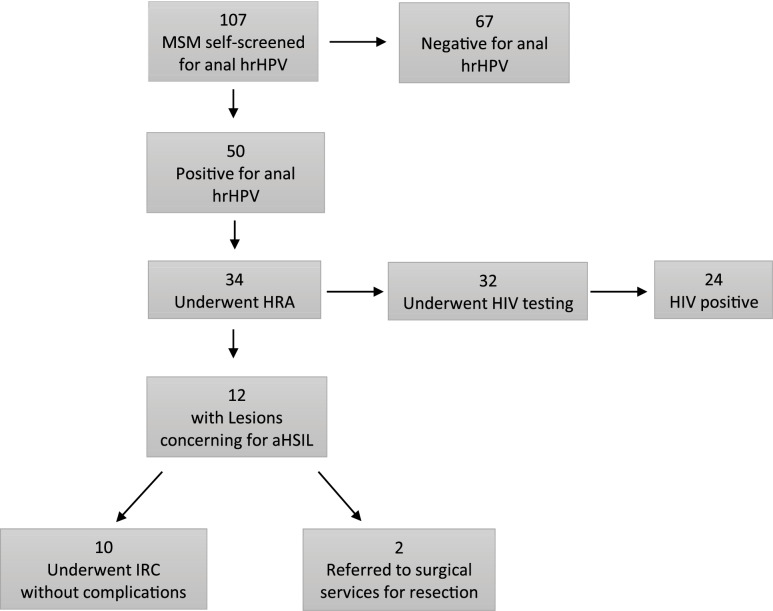
Screen‑triage‑treat management pathway for aHSIL among MSM participants in Liberia. MSM, men who have sex with men; hrHPV, high‑risk human papillomavirus; HRA, high‑resolution anoscopy; HIV, human immunodeficiency virus; aHSIL, atypical high‑grade squamous intraepithelial lesion; IRC, infrared coagulation.

**Figure 2:**
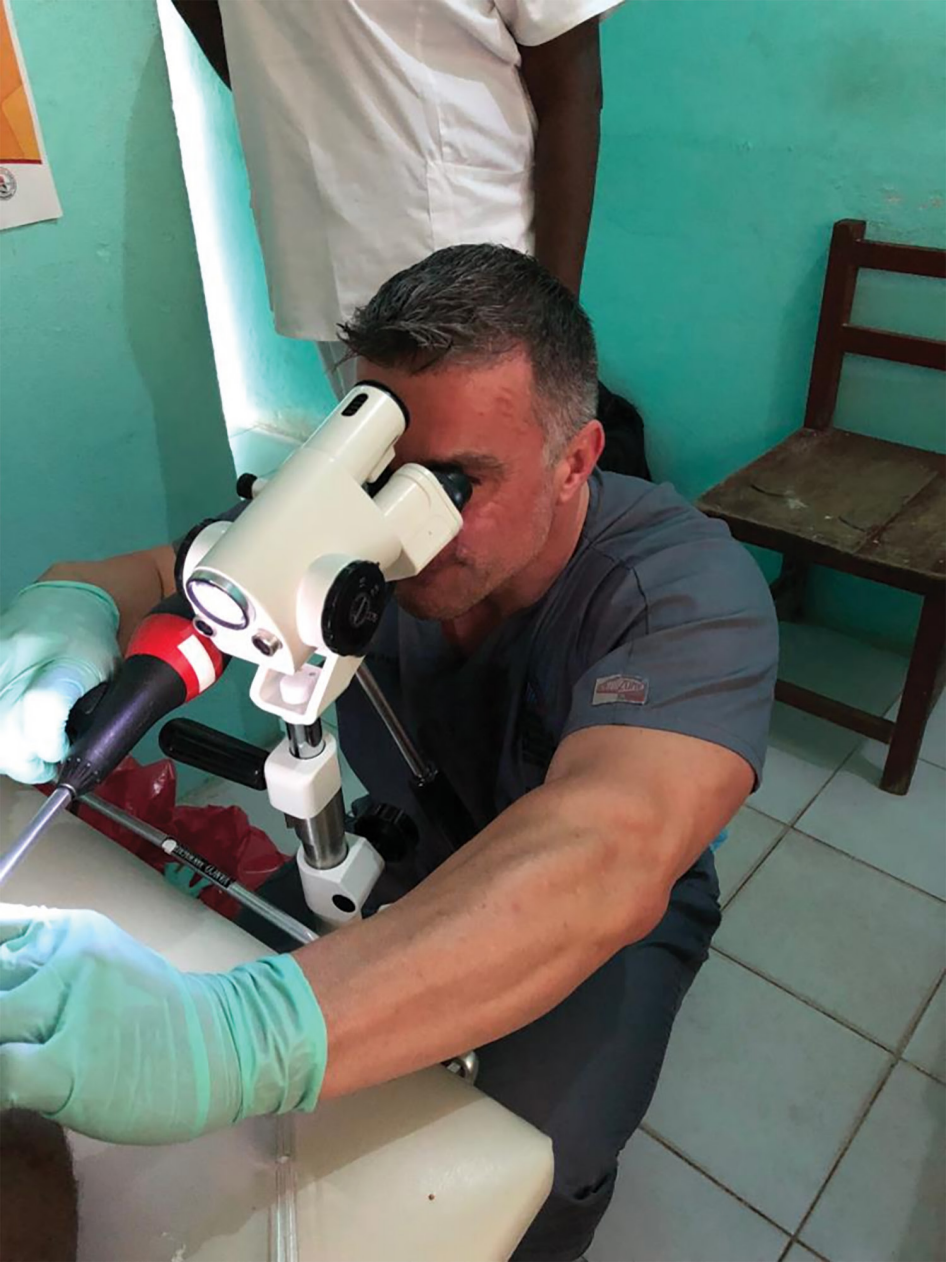
Performance of high‑resolution anoscopy with infrared coagulation. Demonstration of using high‑resolution anoscopy with a magnified anoscope (white instrument) and infrared coagulation (black instrument) to treat anal high‑grade squamous intraepithelial lesions at Redemption Hospital as a method for reducing anal cancer development.

A total of 50 study subjects (45%) tested positive for the presence of anal high‑risk human papillomavirus (hrHPV). Among the 50 MSM who tested positive for hrHPV, 34 participants (68%) elected to undergo high‑resolution anoscopy (HRA; Figure 1). In all, 32 participants (94%) who underwent HRA were screened for HIV, and 24 (75%) tested HIV positive. Among 34 participants who underwent HRA, 12 (35%) were found to have lesions concerning for aHSIL. Of these, 10 participants with lesions concerning for aHSIL (84%) were immediately treated with infrared coagulation, while 2 (16%) had such extensive disease that more invasive surgical intervention was deemed necessary and was performed later by a colorectal surgeon. Overall, treatments were well tolerated without significant immediate or delayed onset adverse reactions other than transient anal discomfort. Figure 2 demonstrates the IRC procedure with typical findings on HRA of benign and aHSIL lesions.

## Discussion

This descriptive study assessed the prevalence of HPV and anal HSIL among MSM in Liberia, and the feasibility of a novel screen‑triage‑treat approach for treating pre‑cancerous lesions. It is the first prospective cohort study of its kind implementing a screen‑triage‑treat model for anal cancer precursors among a high‑risk population in a low‑resource setting. This model involved a screening algorithm whereby the presence of oncogenic anal HPV on self‑swab triggered referral for high‑resolution anoscopy (HRA) and immediate treatment of lesions suspicious for aHSIL. Overall, the model was successful at identifying and treating patients with aHSIL through trusted community peer educators with limited resources to prevent progression to anal cancer. This pilot study provides a feasible framework to identify and treat aHSIL, precancerous lesions, and anal cancer in low‑resource settings.

Our study results demonstrated prevalent HPV infection, aHSIL, and needs for treatment of precancerous lesions among Liberian MSM. Among 107 participants, 45% had prevalent anal HPV infection, and two‑thirds of those underwent HRA. This rate of anal hrHPV is somewhat lower than expected, as rates of hrHPV among MSM populations sampled in other countries is higher, including in the USA (82%), Italy (79.6%), and Mali (80%) [11, 15, 16]. These lower rates could be due to systematic errors, including test performance, specimen handling with international shipping of testing kits from the USA, and transport in‑country between the study site and lab testing facility. Self‑swabbing may have introduced error, as participants were instructed but not supervised during collection. A similar study substituting trained care providers to swab could be illuminating. Finally, SAIL focuses on patient sexual health education and empowerment, such that this population may have lower rates of hrHPV due to increased education and focuses on safer sexual practices. A pooled analysis of nearly 30,000 patients from 64 studies found more comparable rates of hrHPV to our study, specifically among HIV negative (41.2%) and HIV positive (74.3%) patients [17].

Among subjects undergoing HRA, 35% had anal lesions clinically suspicious for anal HSIL. This figure is comparable to what is reported in the literature [17, 18]. Self‑reported rates of HIV among all MSM (19.6%) are higher than prevalence rates in the USA, Europe, and Asia, but are comparable to other rates in sub‑Saharan Africa [19–22]. However, there was a high HIV prevalence among aHSIL‑positive participants, as 94% were tested for HIV with a resultant seroprevalence of 75%, and 20% of the overall sample was HIV positive. Population‑level data suggests a national HIV rate of 1.3% in Liberia, but that only 76% of these individuals know their status [23, 24]. One study conducted at JFK Hospital from 2014 to 2018 among 66,256 patients demonstrated an HIV prevalence of 10%, suggesting that these national level rates may be underestimates [25]. There is even less data on HIV prevalence among other vulnerable subpopulations in Liberia, including transgender persons, sex workers, and queer persons, who may be at increased risk, suggesting the need for increased screening interventions among these populations.

This pilot demonstrated a screening‑triage‑treat model as a successful option for screening and treatment of precancerous and cancerous lesions in a low‑resource environment. Screen‑triage‑treat has been implemented in other settings for the treatment of gynecologic disease, including cervical cancer [26, 27]. While this study makes a strong case for the feasibility of screen‑triage‑treat models for MSM and other high‑risk populations in low‑resource areas, important barriers exist to sustainable implementation and scalability. HRA can be costly and at present requires specialty training that is not available to healthcare workers in Liberia. Efforts directed toward specialty‑level training or procedure‑specific workshops among appropriately trained local healthcare professionals are needed. This study demonstrated significant loss to follow‑up, particularly between those who tested positively for aHSIL and those who underwent HRA (*n* = 16, 32%). Future studies could implement qualitative methodologies among patients and other stakeholders to identify implementation and follow‑up barriers. Peer educators were a significant component that contributed to the successes of this program, and they present a model for high‑impact community‑based participation and empowerment. Future efforts toward scalability could focus on measuring strategies for recruiting, training, and compensating peer educators.

### Limitations

This study has several limitations. First, it contained a small sample size among a specific population of participants, limiting generalizability. Second, the sample recruitment method did not promote randomization, affecting study validity. Though appropriate for this pilot study to recruit participants privately through trusted sources, co‑designing methodologies with local stakeholders that allow for randomization will be important for future studies. Finally, key components of the study are subjected to bias, particularly response bias through the self‑reported demographics and sexual practices survey, and systemic error bias from the HPV diagnostic tests. Conducting similar studies with comparisons to gold‑standard controls could more rigorously validate this study’s approach.

## Conclusion

The screen‑triage‑treat model, with proven success in management of cervical dysplasia, may be a viable option to treat aHSIL to prevent anal cancer in low‑resource settings. Our findings suggest that a screen‑triage‑treat model presents a feasible option to reduce the incidence of anal cancer among MSM in Liberia. The study showed high rates of hrHPV and HIV, the presence of aHSIL, and a need for treatment that was performed successfully with infrared coagulation with minimal adverse outcomes. Given that the burden of disease of anal cancer is higher in MSM, PLWHA, and sex workers, and access to care in LMICs is limited, there are opportunities to implement and scale a screen‑triage‑treat model among vulnerable populations in LMICs. Future studies should focus on improving follow‑up for hrHPV‑positive patients to undergo HRA, scaling a screen‑triage‑treat model for larger and variant populations, and training local healthcare workers to promote the sustainability of this screening and treatment approach.

## Data Availability

Data are available upon reasonable request from the corresponding author.

## Verification Statement

All authors had access to the data and a role in writing the manuscript.
